# Clusters of prediabetes and type 2 diabetes stratify all-cause mortality in a cohort of participants undergoing invasive coronary diagnostics

**DOI:** 10.1186/s12933-023-01923-3

**Published:** 2023-08-17

**Authors:** Katsiaryna Prystupa, Graciela E. Delgado, Angela P. Moissl, Marcus E. Kleber, Andreas L. Birkenfeld, Martin Heni, Andreas Fritsche, Winfried März, Robert Wagner

**Affiliations:** 1 Department of Internal Medicine IV, Division of Endocrinology, Diabetology and Nephrology, University of Tübingen, Tübingen, Germany; 2 Institute for Diabetes Research and Metabolic Diseases of the Helmholtz Center Munich, University of Tübingen, Otfried-Müller-Str. 10, 72076 Tübingen, Germany; 3 German Center for Diabetes Research (DZD), Neuherberg, Germany; 4 Vth Department of Medicine (Nephrology, Hypertensiology, Rheumatology, Endocrinology, Diabetology), Medical Faculty Mannheim, University of Heidelberg, Mannheim, Germany; 5 Center for Preventive Medicine and Digital Health Baden-Württemberg (CPDBW), Medical Faculty Mannheim, Heidelberg University, Mannheim, Germany; 6 Institute of Nutritional Sciences, Friedrich Schiller University Jena, Jena, Germany; 7 Competence Cluster for Nutrition and Cardiovascular Health (nutriCARD) Halle-Jena-Leipzig, Jena, Germany; 8 SYNLAB MVZ für Humangenetik Mannheim GmbH, Mannheim, Germany; 9 Institute for Clinical Chemistry and Pathobiochemistry, Department for Diagnostic Laboratory Medicine, University Hospital Tübingen, Tübingen, Germany; 10 SYNLAB Academy, SYNLAB Holding Deutschland GmbH, Augsburg and Mannheim, Munich, Germany; 11 Department of Endocrinology and Diabetology, Medical Faculty and University Hospital, Heinrich Heine University, Düsseldorf, Germany; 12 Institute for Clinical Diabetology, German Diabetes Center (DDZ), Leibniz Center for Diabetes Research at Heinrich-Heine University, Auf’m Hennekamp 65, 40225 Düsseldorf, Germany; 13 Division of Endocrinology and Diabetology, Internal Medicine 1, University Hospital Ulm, Ulm, Germany

**Keywords:** Clusters, Prediabetes, Type 2 diabetes, All-cause mortality

## Abstract

**Background:**

Heterogeneous metabolic clusters have been identified in diabetic and prediabetic states. It is not known whether such pathophysiologic clusters impact survival in at-risk persons being evaluated for coronary heart disease.

**Methods:**

The LURIC Study recruited patients referred for coronary angiography at a median age of 63 (IQR 56–70) with a follow-up of 16.1 (IQR 9.6, 17.7) years. Clustering of 1269 subjects without diabetes was performed with oGTT-derived glucose and insulin; fasting triglyceride, high-density lipoprotein, BMI, waist and hip circumference. Patients with T2D (n = 794) were clustered using age, BMI, glycemia, homeostasis model assessment, and islet autoantibodies. Associations of clusters with mortality were analysed using Cox regression.

**Results:**

Individuals without diabetes were classified into six subphenotypes, with 884 assigned to subjects at low-risk (cluster 1,2,4) and 385 at high-risk (cluster 3,5,6) for diabetes. We found significantly increased mortality in clusters 3 (hazard ratio (HR)1.42), 5 (HR 1.43), and 6 (HR 1.46) after adjusting for age, BMI, HbA1c and sex. In the T2D group, 508 were assigned to mild age-related diabetes (MARD), 183 to severe insulin-resistant diabetes (SIRD), 84 to mild obesity-related diabetes (MOD), 19 to severe insulin-deficient diabetes (SIDD). Compared to the low-risk non-diabetes group, crude mortality was not different in MOD. Increased mortality was found for MARD (HR 2.2), SIRD (HR 2.2), and SIDD (HR 2.5).

**Conclusions:**

Metabolic clustering successfully stratifies survival even among persons undergoing invasive coronary diagnostics. Novel clustering approaches based on glucose metabolism can identify persons who require special attention as they are at risk of increased mortality.

**Supplementary Information:**

The online version contains supplementary material available at 10.1186/s12933-023-01923-3.

## Introduction

A solely glucose-based definition of prediabetes and type 2 diabetes (T2D) cannot sufficiently account for possible differences in pathophysiology and fails to predict disease progression, complications and therapeutic success. Ahlquist et al. extended the classic T2D definition by subdividing the disease into 5 clusters [1]. This novel clustering approach is based on age at diagnosis, presence of islet autoantibodies, BMI, glycemia, insulin sensitivity and insulin secretion as assessed from fasting C-peptide and glucose levels. The authors proposed 5 different pathophysiological clusters, also highlighting different trajectories of diabetes progression.

We recently applied a similar approach to persons not yet suffering from diabetes. We identified specific constellations of key metabolic variables in persons at increased risk for diabetes [2], and defined 6 distinct clusters of prediabetic metabolism. This clustering procedure can stratify subjects into groups long before prediabetes or T2D occurs.

The grouping techniques in both overt diabetes as well as in prediabetes identify clusters within different trajectories of diabetes development, progression of complications and mortality risk. For example, patients with severely insulin-resistant diabetes are at increased risk of diabetic nephropathy and cardiovascular disease [1, 3, 4], those in prediabetes cluster 6 have an increased risk of kidney disease and all-cause mortality, despite only moderate risk of T2D [2].

However, it is not known whether such pathophysiological clusters of prediabetes and diabetes affect survival even in individuals with clinically suspected cardiovascular disease.

Our study aimed to assign participants of a large cohort who underwent cardiac catheterization to prediabetes and diabetes clusters and investigate overall mortality for the clusters.

## Research design and methods

### Participants

We included participants with newly diagnosed T2D and without diabetes, who were part of the prospective Ludwigshafen Risk and Cardiovascular Health (LURIC) study [5]. The LURIC study aimed to identify individual risk for cardiovascular diseases and to use these results to improve prevention. The study recruited patients with clinically suspected silent or symptomatic coronary disease or acute coronary syndrome (unstable angina and acute myocardial infarction) who underwent coronary angiography [5]. Participants with acute coronary syndrome (ACS) were recruited within a few days after their transfer from intensive/coronary care to the general ward when they were in stable clinical condition. Patients with any acute illness other than ACS, severe non-cardiac chronic disease, as judged by the study physician, or cancer diagnosed within the past five years were excluded. Exclusion criteria were: any acute illness other than ACS, systematically relevant chronic non-cardiac diseases (i.e., chronic renal failure, severe rheumatic arthritis), or a malignant disease diagnosed within five years [5].

A standardised individual and family history questionnaire and comprehensive laboratory testing (including a glucose tolerance test in participants without diabetes) were obtained from all participants. During a mean follow-up of 16.1 (IQR 9.6, 17.7) years, patients or their family physicians were regularly contacted by the study team to assess outcomes [5]. Information on deceased participants was obtained from local population registers.

**Fig. 1:**
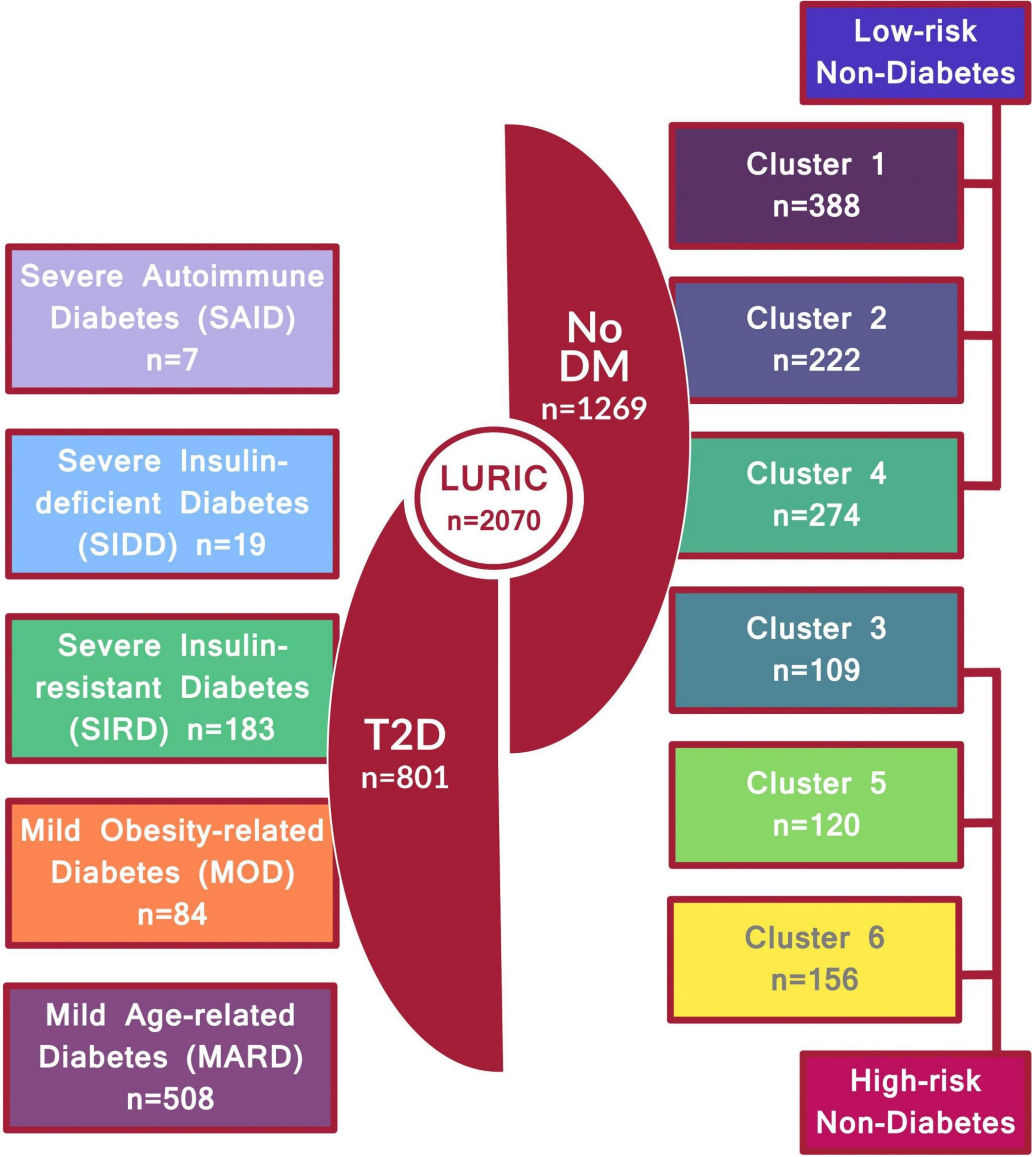
The individuals from the LURIC cohort (n = 2070) were stratified by diabetes status into T2D (n = 801) and non-diabetes (n = 1269). Participants were further grouped into 5 diabetes clusters and 6 non-diabetic clusters, combined into low-risk and high-risk subgroups, which indicate an increased risk of diabetes and metabolic complications

Our analysis included all participants of the LURIC study (N = 3316). We excluded people with type 1 diabetes, T2D duration longer than 5 years [6], insulin-treated patients, or incomplete data of the necessary variables for clustering. This resulted in a subset of 2070 participants (801 with and 1269 without diabetes, Fig. 1). Daily physical activity was documented using an 11-point scale varying from bedrest to very sportive [5, 7]. Key-points on the ranking were 1, bed rest; 2, mostly supine; 3, not very active; 6, usual office work; 9, heavy work or sports; and 11, extremely sportive. In our analyses, we defined physically active participants with a score higher than 6.

### Cohort stratification

The cohort was stratified by diabetes status into T2D (n = 801) and non-diabetes (n = 1269). 1269 participants without diabetes were stratified into prediabetes clusters [2]. The clustering was performed using an online application (http://www.bit.ly/PrediabetesCluster) that, from previously determined cluster centroids, identifies the closest cluster centroid to each participant based on Euclidean distances of the variables glucose and insulin in fasting and at 120 min after oral glucose load; fasting triglyceride, high-density lipoprotein, BMI, waist, and hip circumference. These individuals were classified into six “prediabetes clusters”. A total of 884 subjects were categorized into low-risk clusters: Cluster 1 (n = 388), Cluster 2 (n = 222), and Cluster 4 (n = 274). Additionally, there were 385 subjects in the high-risk clusters, which indicate an increased risk of diabetes and metabolic complications: Cluster 3 (n = 109), Cluster 5 (n = 120), and Cluster 6 (n = 156).

Participants with T2D (N = 801) were stratified into Ahlqvist-clusters using the cluster centroids in the ANDIS cohort derived from age at onset, BMI, HbA1c, HOMA2-B and HOMA2-IR published in the original paper [1, 6]. Cluster membership was determined based on the nearest calculated Euclidean distance to the cluster-defining centroids. This grouping resulted in 508 mild age-related diabetes (MARD), 84 mild obesity-related diabetes (MOD), 183 severe insulin-resistant diabetes (SIRD), 19 severe insulin-deficient diabetes (SIDD) and 7 severe autoimmune diabetes (SAID) who were first classified as type 2 diabetes but had antibodies against glutamic acid decarboxylase. The SAID cluster was excluded from the analysis due to the small number of participants (n = 7). Thus the number of participants in the T2D group decreased to 794. Most participants with diabetes (651 out of 801) had newly diagnosed diabetes. The baseline characteristics for the whole cohort are shown in Table 1. The characteristics of subjects without diabetes and with type 2 diabetes, stratified by clusters, are presented in Supplementary Tables 1 and Supplementary Table 2 respectively.

**Table 1:** Baseline characteristics stratified by diabetes status: Non-Diabetes and Type 2 Diabetes (T2D)

	Non-Diabetes	T2D	p
N	1269	794	
Age (years)	61.03 [53.72, 68.45]	64.91 [58.03, 71.48]	< 0.001
Body mass index (kg/m2)	26.73 [24.47, 29.19]	27.86 [25.35, 30.55]	< 0.001
Sex = F (%)	347 ( 27.3)	222 (28.0)	0.703
Glycosylated hemoglobin (%)	5.70 [5.40, 6.00]	6.60 [6.10, 7.10]	< 0.001
Hypertension = yes (%)	1168 ( 92.0)	765 (96.3)	< 0.001
Antihypertensive medication = yes (%)	1064 ( 83.8)	714 (89.9)	< 0.001
Dyslipidaemia = yes (%)	796 ( 62.7)	598 (75.3)	0.109
Cholesterol-lowering medication = yes (%)	574 ( 45.2)	390 (49.1)	< 0.001
All-cause death = yes (%)	498 ( 39.2)	488 (61.5)	< 0.001
Triglycerides (mg/dl)	133.00 [102.00, 186.00]	158.00 [119.00, 216.00]	< 0.001
LDL cholesterol (mg/dL)	117.00 [99.00, 140.00]	114.00 [92.00, 137.00]	0.016
HDL cholesterol (mg/dL)	39.00 [33.00, 47.00]	36.00 [30.00, 42.00]	< 0.001
Lactate dehydrogenase (U/L)	163.00 [144.00, 186.00]	173.00 [152.00, 200.00]	< 0.001
Iron (µg/dl)	94.00 [72.00, 119.00]	87.00 [65.00, 113.00]	< 0.001
Transferrin (mg/dl)	247.00 [226.00, 273.00]	253.00 [227.00, 277.75]	0.006
Ferritin (ng/ml)	149.00 [86.00, 257.00]	186.00 [106.00, 307.75]	< 0.001
Total protein (g/dl)	6.90 [6.50, 7.20]	6.90 [6.50, 7.20]	0.066
Albumin (g/dl)	4.30 [4.00, 4.80]	4.30 [4.00, 4.70]	0.126
Total bilirubin (mg/dl)	0.60 [0.40, 0.80]	0.50 [0.40, 0.80]	0.986
Amylase (U/L)	19.00 [15.00, 24.00]	18.00 [14.00, 23.00]	0.043
Alkaline phosphatase (U/L)	110.00 [92.00, 131.00]	118.00 [97.00, 143.00]	< 0.001
γ-Glutamyl-transferase (U/L)	15.00 [10.00, 26.00]	19.00 [12.00, 32.00]	< 0.001
Cholinesterase (U/L)	5710.00 [4910.00, 6530.00]	5710.00 [4750.00, 6620.00]	0.878
Creatine kinase (U/L)	30.00 [22.00, 43.00]	28.00 [19.00, 40.00]	0.001
Cortisol (mg/L)	20.90 [17.10, 25.60]	22.15 [17.70, 26.50]	0.001
Aldosterone (ng/L)	79.00 [48.00, 126.00]	78.00 [46.00, 122.00]	0.659
Renin (U/L)	17.00 [9.00, 34.00]	22.00 [10.00, 47.00]	< 0.001
Folic acid (µg/L)	7.60 [5.80, 10.00]	7.90 [6.10, 10.20]	0.088
Cotinine > 15 µg/L = yes (%)	196 ( 15.4)	122 (15.4)	1.000
eGFR CKD-EPI*	89.69 [77.83, 98.41]	86.65 [72.15, 95.71]	< 0.001
Coronary artery disease (CAD) by angiographic status, n (%)			
Normal (smooth contours)	332 (26.5)	138 ( 17.7)	< 0.001
Minor disease (11–49%)	133 (10.6)	70 ( 9.0)	
1 vessel disease (≥ 50%)	236 (18.8)	149 ( 19.1)	
2 vessel disease (≥ 50%)	223 (17.8)	172 ( 22.1)	
3 vessel disease (≥ 50%)	330 (26.3)	251 ( 32.2)	
≥ 10% max. stenosis	974 (77.7)	683 ( 87.6)	< 0.001
≥ 20% max. stenosis	922 (73.5)	642 ( 82.3)	< 0.001
≥ 50% max. stenosis	789 (62.9)	572 ( 73.3)	< 0.001

* Kidney function was estimated using the Chronic Kidney Disease Epidemiology Collaboration (CKD-EPI) equation to calculate the glomerular filtration rate (eGFR).

### Statistical analysis

Baseline demographics of subjects classified by diabetes status and subphenotypes are described as percentages for categorical data and continuous data are presented as median and IQR.

Comparisons between groups were performed using the Chi-squared test for categoric data and ANOVA for continuous data. Kaplan-Meier curves with the log-rank test were used to evaluate differences in overall mortality for the groups The survival probability is calculated as the number of subjects surviving divided by the number of patients at risk.

Hazard ratios (HR) for mortality categories were calculated using Cox proportional hazards regression models, which were adjusted for potential confounding variables. The proportionality assumption test for cox models was assessed by Schoenfeld residuals.

In the reported analyses, model 1 describes the crude association, model 2 was adjusted for age, sex, BMI, glycosylated haemoglobin (HbA1c) and model 3 also corrected for smoking, physical activity, antihypertensive medication, lipid-lowering medication, antidiabetic medication, anti-platelets/anti-coagulant therapy and severity of coronary artery disease based on angiography. Associations were considered as significant at a p-value of < 0.05. All calculations were performed using R version 4.0.3 and the following packages were utilized: ‘stats’, ‘tableone’, ‘survival’, ‘survminer’, ‘tidyverse’ [8].

## Results

### Stratification of all-cause mortality in participants without diabetes

Characteristics of all analysed subjects without diabetes are summarised in Table 1.

Crude mortality was not different among clusters using the Kaplan-Meier estimator with a log-rank test p = 0.4 (Supplementary Fig. 1). The unadjusted Cox regression analysis revealed that only cluster 3 was associated with an increased risk of death compared to cluster 1, with the hazard ratio (HR) = 1.40, CI:1.02–1.93, p = 0.037. In model 2, with adjustments for age, BMI, sex and HbA1c, higher mortality was found for cluster 3 (HR = 1.42, CI:1.024–1.97, p = 0.036), cluster 5 (HR = 1.43, CI:1.01–2.03, p = 0.043) and cluster 6 (HR = 1.46, CI:1.05–2.03, p = 0.024). The correction for a range of conventional risk factors in model 3 showed a significantly elevated hazard in cluster 3 (HR = 1.40, CI:1.00-1.95, p = 0.048) and cluster 6 (HR = 1.46, CI:1.04–2.04, p = 0.027) compared to cluster 1 (Supplementary Fig. 3). At the same time, current smoking, biochemically assessed by elevated plasma cotinine level over 15 µg/L [5, 9], demonstrated a strong association with an increased risk of all-cause mortality (HR = 2.62, CI:2.02–3.38, p < 0.001).

In turn, physical exercise contributes to lower mortality by almost 30%, showing HR = 0.73, CI:0.61–0.88 with p < 0.001 in a group of non-diabetes.

### Stratification of all-cause mortality in participants with type 2 diabetes

The MOD cluster comprises younger participants with higher BMI, SIDD has markedly increased HbA1c and the MARD cluster comprises the oldest participants with relatively lower BMI.

The Kaplan-Meier curves revealed different survival probabilities across T2D clusters (p = 0.0011, log-rank test; Supplementary Fig. 2). Mortality in non-adjusted Cox regression was significantly higher in SIDD (HR = 2.34, CI:1.25 to 4.4, p = 0.008), SIRD (HR = 2.04, CI:1.4 to 3.0, p = 0.0004) and MARD (HR = 2.03, CI: 1.4 to 2.9, p = 0.0001) compared to MOD cluster. Using models 2 and 3, no statistically significant difference in mortality was found for clusters SIDD, SIRD and MARD compared to cluster MOD. Physical activity was associated with a decreased (HR = 0.62, CI:0.52 to 0.75, p < 0.001) all-cause mortality, whereas active nicotine use increased mortality by 90% (HR = 1.90, CI:1.46 to 2.47, p < 0.001).

### Stratification of all-cause mortality in the entire cohort

We pooled subjects with and without diabetes to analyse differences in all-cause mortality across the whole spectrum of glucose metabolism states from prediabetes to diabetes. Patients without diabetes were combined into low-risk (clusters 1,2,4) and high-risk (clusters 3,5,6) groups, which indicate an increased risk of diabetes and metabolic complications.

**Fig. 2:**
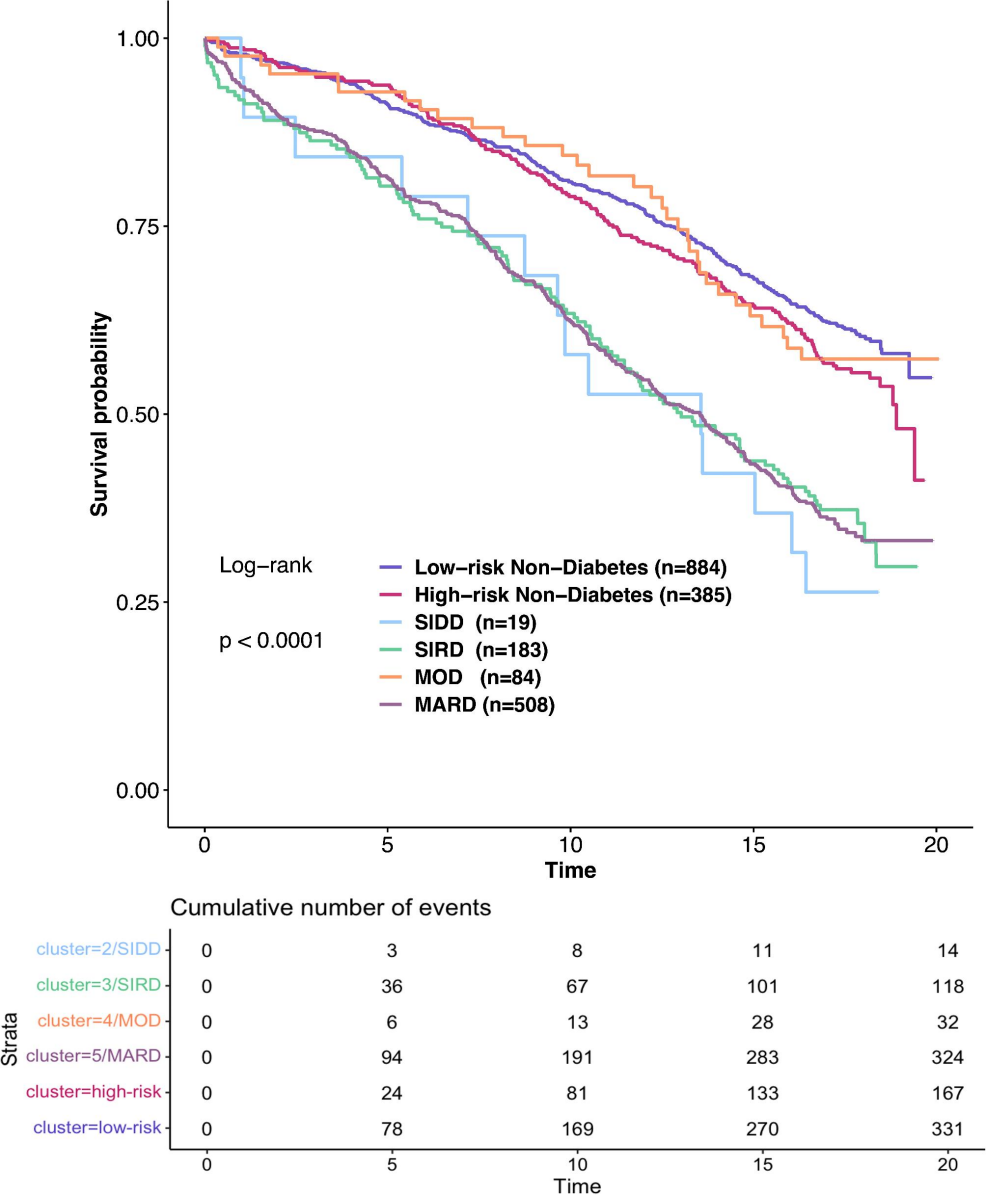
Kaplan-Meier plots for all-cause mortality according to non-diabetes clusters: high- and low-risk groups of diabetes and metabolic complications; and T2D clusters: Severe Insulin-Deficient Diabetes (SIDD), Severe Insulin-Resistant Diabetes (SIRD), Mild Obesity-Related Diabetes (MOD), Mild Age-Related Diabetes (MARD)(numbers in columns represent medians and interquartile ranges). Log-rank tests revealed significant differences among T2D clusters (p < 0.0001)

The Kaplan-Meier curves show different mortality rates across the analysed groups (p < 0.0001, Fig. 2). Three novel diabetes clusters, mainly SIRD, MARD and SIDD, showed higher mortality than MOD and non-diabetes groups.

In unadjusted Cox models with the low-risk non-diabetes group as reference, we found higher mortality risk for SIDD (HR = 2.52, CI:1.48–4.3, p < 0.001), SIRD and MARD (HR = 2.18, CI:1.77–2.69 and HR = 2.17, CI:1.86–2.53, respectively, both p-values < 0.0001).

**Fig. 3:**
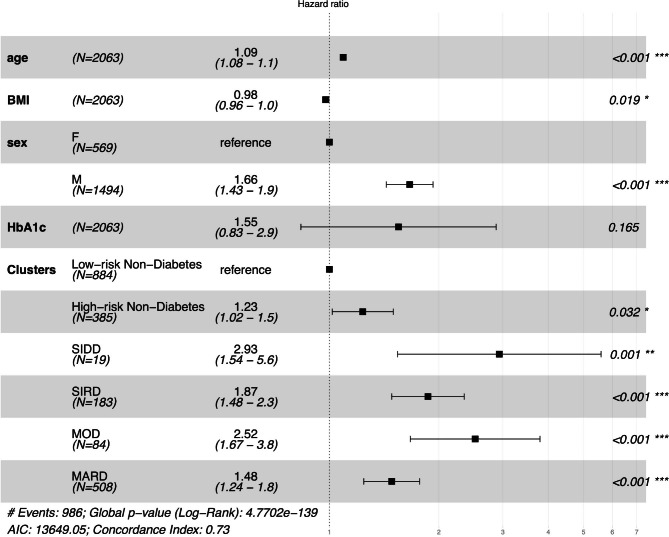
Forest Plot for Cox proportional hazards **model 2** for the entire cohort stratified into clusters: low-risk and high-risk of diabetes and metabolic complications groups, Severe Insulin-Deficient Diabetes (SIDD), Severe Insulin-Resistant Diabetes (SIRD), Mild Obesity-Related Diabetes (MOD), Mild Age-Related Diabetes (MARD).

After adjustments for age, BMI, sex and HbA1c, all clusters have significantly higher mortality compared to the low-risk non-diabetes group (Fig. 3). The high-risk non-diabetes cluster has over 25% higher hazards than the low-risk non-diabetes clusters (HR = 1.26, CI:1.03–1.52, p < 0.021). The MARD cluster has the lowest hazard ratio for all-cause mortality across diabetes subgroups (HR = 1.49, CI:1.25–1.77, p < 0.001). Additional adjustments in model 3 revealed significantly elevated mortality hazard only in diabetes clusters compared to the low-risk prediabetes cluster.

## Discussion

In this study, we investigated differences in all-cause mortality across previously defined clusters of diabetes and prediabetes. These groups were identified in a high-risk cohort comprising patients undergoing coronary angiography [5]. The key finding is that metabolic clustering stratifies mortality even in a cohort enriched with patients having coronary artery disease.

Among clusters without diabetes, clusters 3, 5 and 6 delineate groups with increased diabetes risk. In the initial work that established these clusters, subjects in cluster 3 had higher cardiovascular risk as indicated by increased carotid intima media thickness [2]. In the current work, cluster 3 showed higher crude mortality than other non-diabetes clusters. After adjustment for sex, age, BMI and HbA1c, all clusters with increased diabetes risk (i.e. clusters 3, 5 and 6) showed elevated all-cause mortality in the current analysis. The cluster characteristics are consistent with the features in the original cohort [2]. For example, clusters 5 and 6 had marked insulin resistance and an impaired lipid profile. Dyslipidaemia and insulin resistance, as well as a combination of these factors, are known to increase cardiovascular mortality [10]. Of note, the prediabetes cluster 6 with only modestly elevated diabetes risk had a similar mortality to the high diabetes risk clusters 3 and 5. This strengthens the concept that mortality in one of the high-risk metabolic clusters before diabetes manifestation is dissociated from the risk of glycaemic progression. Consistent with the findings that the SIRD cluster and cluster 6 have an increased risk of nephropathy, we observe lower eGFR in the cross-sectional data in these groups.

In addition, we here show that also the Ahlqvist-clusters stratify mortality among patients with overt T2D. Without accounting for anthropometric differences across clusters, the obesity-related cluster showed lower mortality than the other diabetes clusters. The MOD-associated mortality was not different from persons without diabetes, whereas the clusters SIDD, SIRD and MARD had significantly higher mortality than MOD. In line, recent studies reported relatively lower cardiovascular mortality in persons of the MOD cluster [11] and fewer micro- and macrovascular complications than other clusters [12]. In our current study, the MOD-cluster comprises young participants with the highest BMI but only moderate insulin resistance compared to SIRD. The combination of these factors in the MOD cluster resembles a phenotype previously described as a metabolically healthy obesity [13, 14]. This can potentially explain the lower mortality in persons assigned to the MOD cluster.

Despite a previously described nominally higher cardiovascular risk in the SIRD cluster compared with the MARD cluster in a recent study [4], crude all-cause mortality in SIRD was not different from MARD or SIDD in our work. Of note, the number of patients in the SIDD cluster was considerably low. The different distribution of diabetes subphenotypes from previous reports [1, 6] most likely results from the specific characteristics of the LURIC study. Higher proportion of older participants (64.9 years, SD: 9.3) in the newly diagnosed T2D group probably explains the prevalence of MARD (mean age was lower in the ANDIS cohort at 60.93 years, SD: 12.25.

The difference in mortality across clusters changed after adjusting for cluster determinants such as age, BMI and HbA1c. This is not unexpected given the crucial importance of these variables in the clustering procedure. Independent from these variables, MARD had the lowest mortality among diabetes clusters compared to the low-risk-non-diabetes group (Fig. 3). These results are consistent with multiple reports that indicated that younger rather than older age at diabetes diagnosis indicates an especially high mortality risk [15]. Hence, age-related type 2 diabetes is associated with lower mortality, independent of the participants’ age, even in our cohort of patients undergoing invasive coronary diagnostics.

In sum, we show that assignment of persons with suspected coronary heart disease to previously defined prediabetes and diabetes clusters also segregates groups with different mortality. Risk stratification by assignment of individuals to discrete clusters instead of using continuous variables in risk models has some weaknesses [16]. However, clusters allow treating combinations of risk variables in groups, and thereby allowing interactions between variables [17]. Also, clusters can be interpretable, clinically meaningful and allow treatment decisions. They can also link known disease features to physiological mechanisms [18].

Limitations of the current work comprise a low participant number in cluster SIDD, lack of ethnic heterogeneity in the study population and the lack of precise data on the cause of death, which limited the analysis of cause-specific mortality.

To our knowledge, this is the first work comprehensively analysing all-cause mortality across prediabetes- and diabetes clusters during a long follow-up. The novel clustering methods can identify persons with an increased risk of premature death. Thus, recognition of distinct metabolic groups can identify persons who require special attention.

### Electronic supplementary material

Below is the link to the electronic supplementary material.

Supplementary Material 1

## Data Availability

The data are not publicly available due to them containing information that could compromise research participant privacy/consent.
