# Supplementary material for: Clusters of prediabetes and type 2 diabetes stratify all-cause mortality in a cohort of participants undergoing invasive coronary diagnostics
Source: Cardiovasc Diabetol. 2023 Aug 17;22:211. doi: 10.1186/s12933-023-01923-3 (PMC10436494; doi:10.1186/s12933-023-01923-3)
Supplement: Supplementary file 1 — Supplementary Material 1 [file 12933_2023_1923_MOESM1_ESM.docx]

Supplementary Materials


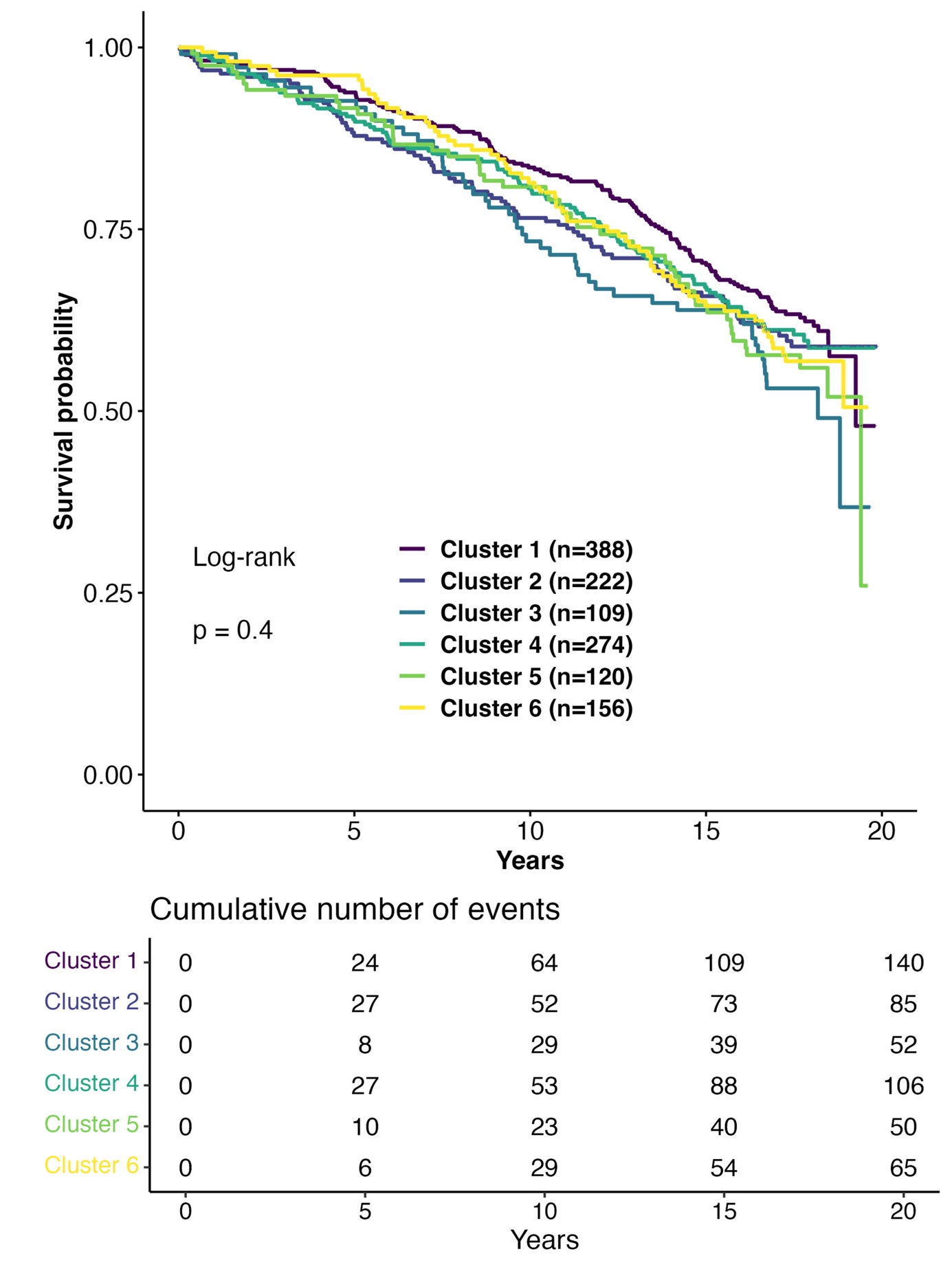


**Supplementary Figure 1:** Kaplan-Meier plots for all-cause mortality according to non-diabetes clusters. Log-rank tests revealed no significant differences among clusters(p=0.4).


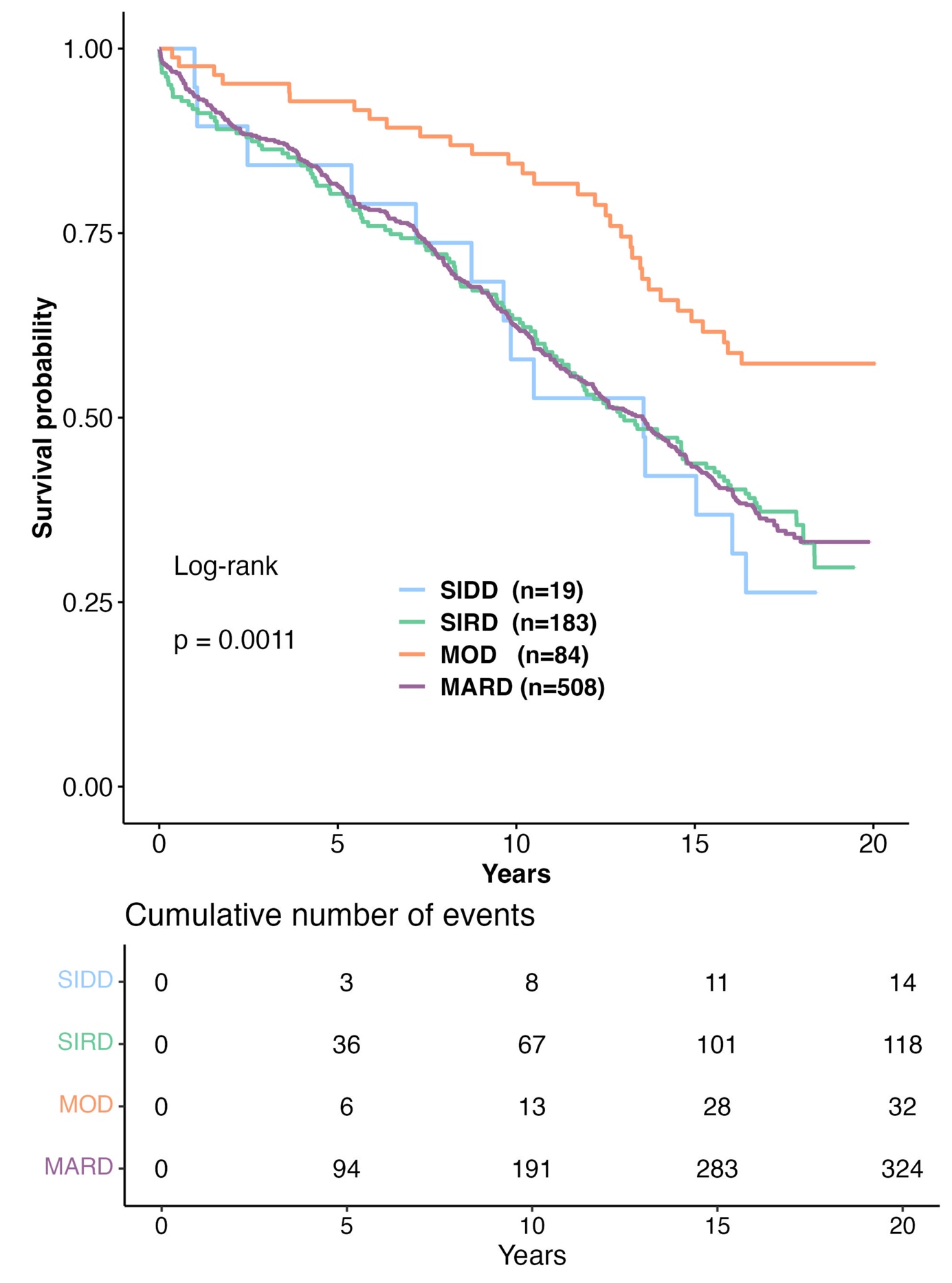


**Supplementary Figure 2:** Kaplan-Meier plots for all-cause mortality according to T2D clusters: Severe Insulin-Deficient Diabetes (SIDD), Severe Insulin-Resistant Diabetes (SIRD), Mild Obesity-Related Diabetes (MOD), Mild Age-Related Diabetes (MARD)(numbers in columns represent medians and interquartile ranges). Log-rank tests revealed significant differences among T2D clusters (p=0.0011).

**Supplementary Figure 3:** Forest Plot for Cox proportional hazards **model 3** for non-diabetes cohort divided into clusters. (CAD- Coronary artery disease)

# **Supplementary Table 1:** Participant characteristics stratified by non-diabetes clusters

|  | 1 | 2 | 3 | 4 | 5 | 6 | p |
| --- | --- | --- | --- | --- | --- | --- | --- |
| N | 388 | 222 | 109 | 274 | 120 | 156 |  |
| Age | 60.40  [53.34, 68.21] | 59.24  [51.54, 66.49] | 62.74  [56.65, 70.66] | 62.20  [54.66, 68.65] | 61.65  [54.67, 69.11] | 60.97  [51.40, 68.82] | 0.017 |
| Body mass index | 25.15  [23.86, 26.86] | 23.92  [22.31, 25.39] | 27.04  [25.71, 28.73] | 28.56  [26.97, 30.54] | 29.06  [26.67, 32.31] | 30.35  [28.37, 32.58] | <0.001 |
| Sex = F (%) | 98 (25.3) | 61 (27.5) | 32 (29.4) | 81 (29.6) | 37 (30.8) | 38 (24.4) | 0.671 |
| Hba1c | 5.70  [5.38, 6.00] | 5.70  [5.30, 5.97] | 5.90  [5.60, 6.20] | 5.70  [5.40, 6.00] | 5.80  [5.50, 6.10] | 5.75  [5.40, 6.00] | <0.001 |
| Hypertension = yes (%) | 352 (90.7) | 192 (86.5) | 102 (93.6) | 257 (93.8) | 119 (99.2) | 146 (93.6) | 0.001 |
| Antihypertensive medication = yes (%) | 330 (85.1) | 166 (74.8) | 90 (82.6) | 238 (86.9) | 112 (93.3) | 128 (82.1) | <0.001 |
| Dyslipidaemia = yes (%) | 257 (66.2) | 62 (27.9) | 67 (61.5) | 186 (67.9) | 111 (92.5) | 113 (72.4) | <0.001 |
| Cholesterol-lowering medication = yes (%) | 199 (51.3) | 74 (33.3) | 43 (39.4) | 117 (42.7) | 65 (54.2) | 76 (48.7) | <0.001 |
| Death = yes (%) | 140 (36.1) | 85 (38.3) | 52 (47.7) | 106 (38.7) | 50 (41.7) | 65 (41.7) | 0.336 |
| Triglycerides (mg/dl) | 136.00 [102.00, 184.25] | 103.00 [81.25, 131.75] | 147.00 [110.00, 186.00] | 129.50 [103.00, 179.00] | 221.50 [161.50, 315.25] | 149.50 [113.00, 196.75] | <0.001 |
| HDL cholesterol (mg/dl) | 38.00 [32.00, 45.00] | 49.00 [42.00, 57.00] | 41.00 [35.00, 47.00] | 38.00 [33.00, 44.00] | 32.50 [27.00, 37.00] | 38.00 [32.00, 45.00] | <0.001 |
| LDL cholesterol (mg/dL) | 117.00 [99.00, 139.00] | 118.00 [100.00, 139.75] | 122.00 [102.00, 147.00] | 119.00 [100.00, 143.00] | 109.00 [88.00, 132.00] | 114.50 [96.75, 142.00] | 0.016 |
| Lactate dehydrogenase (U/L) | 158.50 [139.00, 180.00] | 156.00 [141.00, 180.00] | 163.00 [145.00, 184.00] | 167.00 [144.25, 194.00] | 167.00 [149.00, 189.50] | 173.00 [151.00, 195.25] | <0.001 |
| Hemoglobin (g/dl) | 14.00 [13.00, 15.00] | 13.80 [13.00, 14.60] | 13.70 [13.00, 14.60] | 13.90 [13.10, 15.00] | 14.10 [13.00, 15.00] | 14.30 [13.70, 15.10] | <0.001 |
| Erythrocytes (pl) | 4.62 [4.24, 4.91] | 4.49 [4.23, 4.76] | 4.54 [4.29, 4.78] | 4.58 [4.30, 4.85] | 4.62 [4.26, 4.86] | 4.69 [4.41, 4.96] | 0.001 |
| Iron (µg/dl) | 89.00 [69.50, 112.00] | 105.50 [77.50, 128.75] | 94.00 [70.00, 121.00] | 96.50 [76.00, 125.00] | 88.00 [69.25, 108.00] | 91.00 [71.75, 114.00] | 0.001 |
| Transferrin (mg/dl) | 249.00 [225.00, 270.25] | 242.50 [222.25, 270.75] | 244.00 [227.00, 267.00] | 247.00 [223.25, 271.00] | 255.00 [231.75, 277.50] | 252.00 [231.00, 285.25] | 0.025 |
| Ferritin (ng/ml) | 150.50 [90.75, 243.00] | 112.50 [65.50, 195.25] | 157.00 [93.00, 294.00] | 137.50 [81.25, 257.25] | 187.00 [104.75, 297.50] | 175.00 [108.75, 306.75] | <0.001 |
| Total protein (g/dl) | 6.80 [6.50, 7.10] | 6.80 [6.50, 7.10] | 6.90 [6.50, 7.20] | 6.80 [6.40, 7.10] | 6.95 [6.60, 7.30] | 7.00 [6.70, 7.30] | <0.001 |
| Albumin (g/dl) | 4.30 [4.00, 4.80] | 4.40 [4.00, 4.80] | 4.40 [4.07, 4.80] | 4.40 [4.07, 4.80] | 4.30 [4.00, 4.70] | 4.30 [4.10, 4.70] | 0.879 |
| Total bilirubin (mg/dl) | 0.50 [0.40, 0.70] | 0.60 [0.40, 0.80] | 0.60 [0.40, 0.80] | 0.60 [0.40, 0.80] | 0.50 [0.40, 0.70] | 0.50 [0.40, 0.70] | 0.013 |
| Amylase (U/L) | 20.00 [15.50, 24.00] | 20.50 [16.00, 25.00] | 19.00 [15.00, 24.00] | 18.00 [14.00, 23.00] | 17.50 [14.00, 22.00] | 18.00 [14.00, 22.25] | <0.001 |
| Alkaline phosphatase (U/L) | 109.00 [90.75, 131.00] | 108.00 [83.25, 128.00] | 107.00 [94.00, 125.00] | 110.00 [93.25, 136.50] | 113.00 [95.00, 140.00] | 117.50 [102.75, 135.25] | <0.001 |
| γ-Glutamyl-transferase (U/L) | 14.00 [10.00, 25.00] | 12.00 [8.00, 22.75] | 17.00 [11.00, 26.00] | 15.00 [10.00, 25.00] | 19.00 [14.00, 32.00] | 18.00 [12.75, 32.00] | <0.001 |
| Cholinesterase (U/L) | 5690.00 [4857.50, 6440.00] | 5450.00 [4595.00, 6305.00] | 5490.00 [4760.00, 6240.00] | 5670.00 [4950.00, 6467.50] | 5975.00 [5150.00, 6940.00] | 6120.00 [5340.00, 7030.00] | <0.001 |
| Creatine kinase (u/l) | 30.00 [21.00, 39.25] | 29.00 [22.00, 41.00] | 29.00 [22.00, 39.00] | 30.00 [20.00, 43.00] | 29.00 [21.75, 42.00] | 38.00 [25.75, 55.00] | <0.001 |
| Cortisol (mg/L) | 21.05 [17.20, 25.20] | 21.30 [17.20, 25.58] | 21.90 [17.90, 27.30] | 20.25 [16.02, 25.58] | 20.50 [17.50, 25.33] | 20.65 [16.35, 25.00] | 0.364 |
| Aldosterone (ng/L) | 76.00 [45.00, 123.25] | 77.00 [51.00, 120.75] | 89.50 [50.50, 133.25] | 79.00 [50.00, 117.75] | 78.00 [47.00, 140.00] | 83.00 [50.00, 139.00] | 0.486 |
| Renin (U/L) | 16.50 [9.00, 36.00] | 15.00 [9.00, 31.00] | 17.00 [7.00, 31.00] | 15.00 [8.00, 29.75] | 18.00 [9.00, 40.25] | 22.00 [12.00, 39.50] | 0.005 |
| Folic acid (µg/L) | 7.50 [5.80, 9.60] | 7.85 [5.80, 10.47] | 8.50 [6.70, 10.70] | 7.70 [6.10, 9.78] | 7.10 [5.80, 10.85] | 6.95 [5.50, 9.43] | 0.031 |
| Cotinine >15µg/L = yes (%) | 61 (15.7) | 48 (21.6) | 11 (10.1) | 38 (13.9) | 17 (14.2) | 21 (13.5) | 0.074 |
| HOMA 2 IR | 1.69 [1.15, 2.17] | 1.25 [0.84, 1.63] | 1.71 [1.05, 2.13] | 1.54 [0.97, 1.94] | 2.41 [1.72, 3.57] | 2.56 [1.76, 3.15] | <0.001 |
| eGFR CKD-EPI* | 89.45 [76.94, 98.41] | 92.47 [83.42, 100.11] | 90.15 [77.50, 98.90] | 90.88 [79.46, 98.10] | 88.53 [71.35, 97.96] | 86.94 [74.35, 97.60] | <0.001 |
| Coronary artery disease (CAD) by angiographic status, n (%) |  |  |  |  |  |  |  |
| Normal (smooth contours) | 94 ( 24.6) | 75 ( 34.1) | 29 ( 27.1) | 79 ( 29.0) | 18 ( 15.1) | 37 ( 24.0) | 0.007 |
| Minor disease (11-49%) | 32 ( 8.4) | 21 ( 9.5) | 10 ( 9.3) | 37 ( 13.6) | 12 ( 10.1) | 21 ( 13.6) |  |
| 1 vessel disease (≥ 50%) | 69 ( 18.1) | 49 ( 22.3) | 22 ( 20.6) | 46 ( 16.9) | 25 ( 21.0) | 25 ( 16.2) |  |
| 2 vessel disease (≥ 50%) | 71 ( 18.6) | 37 ( 16.8) | 15 ( 14.0) | 47 ( 17.3) | 21 ( 17.6) | 32 ( 20.8) |  |
| 3 vessel disease (≥ 50%) | 116 ( 30.4) | 38 ( 17.3) | 31 ( 29.0) | 63 ( 23.2) | 43 ( 36.1) | 39 ( 25.3) |  |
| ≥ 10% max. Stenosis | 307 ( 80.4) | 150 ( 68.2) | 84 ( 78.5) | 203 ( 74.6) | 103 ( 86.6) | 127 ( 82.5) | 0.001 |
| ≥ 20% max. Stenosis | 288 ( 75.4) | 145 ( 65.9) | 78 ( 72.9) | 193 ( 71.0) | 101 ( 84.9) | 117 ( 76.0) | 0.005 |
| ≥ 50% max. Stenosis | 256 ( 67.0) | 124 ( 56.4) | 68 ( 63.6) | 156 ( 57.4) | 89 ( 74.8) | 96 ( 62.3) | 0.003 |

* Kidney function was estimated using the Chronic Kidney Disease Epidemiology Collaboration (CKD-EPI) equation to calculate the glomerular filtration rate (eGFR).

| Supplementary Table 2: Participant characteristics stratified by T2D clusters according to Ahlqvist et al.: Severe Insulin-Deficient Diabetes (SIDD), Severe Insulin-Resistant Diabetes (SIRD), Mild Obesity-Related Diabetes (MOD), Mild Age-Related Diabetes (MARD) | | | | | |
| --- | --- | --- | --- | --- | --- |
|  | **2/SIDD** | **3/SIRD** | **4/MOD** | **5/MARD** | **p** |
| n | 19 | 183 | 84 | 508 |  |
| Age | 60.20 [56.75, 65.92] | 64.24 [58.61, 70.44] | 52.41 [47.08, 58.03] | 67.37 [60.98, 72.77] | <0.001 |
| BMI | 29.41 [26.45, 30.76] | 29.40 [27.18, 32.24] | 32.27 [29.61, 34.73] | 26.87 [24.58, 28.85] | <0.001 |
| Sex = F (%) | 8 ( 42.1) | 44 ( 24.0) | 17 ( 20.2) | 153 ( 30.1) | 0.074 |
| HbA1c | 10.60 [9.65, 11.30] | 6.50 [6.00, 6.80] | 6.80 [6.50, 7.40] | 6.60 [6.10, 7.03] | <0.001 |
| Hypertension = yes (%) | 18 ( 94.7) | 178 ( 97.3) | 81 ( 96.4) | 488 ( 96.1) | 0.873 |
| Antihypertensive medication = yes (%) | 18 ( 94.7) | 172 ( 94.0) | 72 ( 85.7) | 452 ( 89.0) | 0.113 |
| Dyslipidaemia = yes (%) | 14 ( 73.7) | 153 ( 83.6) | 74 ( 88.1) | 357 ( 70.3) | <0.001 |
| Cholesterol-lowering medication = yes (%) | 12 ( 63.2) | 93 ( 50.8) | 47 ( 56.0) | 238 ( 46.9) | 0.228 |
| Death = yes (%) | 14 ( 73.7) | 118 ( 64.5) | 32 ( 38.1) | 324 ( 63.8) | <0.001 |
| HDL cholesterol (mg/dL) | 36.00 [30.00, 42.00] | 34.00 [28.00, 40.00] | 34.00 [29.00, 38.00] | 37.00 [31.00, 43.00] | <0.001 |
| LDL cholesterol (mg/dL) | 108.21 (32.51) | 111.11 (32.66) | 113.72 (45.56) | 118.62 (32.98) | 0.048 |
| Triglycerides (mg/dL) | 181.00 [142.50, 315.00] | 179.00 [130.00, 247.50] | 172.00 [135.50, 267.00] | 150.00 [110.75, 202.00] | <0.001 |
| Lactate dehydrogenase (U/L) | 173.00 [151.00, 200.50] | 175.00 [152.50, 209.00] | 174.00 [154.00, 191.50] | 171.00 [150.00, 199.00] | 0.465 |
|  |  |  |  |  |  |
| HOMA2IR | 2.00 [1.18, 3.56] | 3.58 [2.79, 4.66] | 2.22 [1.38, 2.68] | 1.57 [0.87, 2.24] | <0.001 |
| Iron (Âµg/dl) | 83.00 [61.00, 103.00] | 86.00 [60.00, 115.00] | 97.00 [77.50, 119.00] | 85.50 [65.00, 112.00] | 0.062 |
| Transferrin (mg/dl) | 254.00 [225.50, 270.50] | 250.00 [226.00, 273.00] | 263.00 [243.75, 295.00] | 253.00 [226.00, 279.00] | 0.056 |
| Ferritin (ng/ml) | 277.00 [189.00, 407.00] | 194.00 [107.50, 325.50] | 243.00 [150.50, 373.25] | 165.00 [98.00, 287.75] | <0.001 |
| Total protein (g/dl) | 6.80 [6.70, 7.00] | 6.90 [6.50, 7.30] | 7.00 [6.68, 7.20] | 6.85 [6.50, 7.20] | 0.734 |
| Albumin (g/dl) | 4.30 [3.90, 4.70] | 4.20 [4.00, 4.50] | 4.30 [4.05, 4.60] | 4.40 [4.00, 4.70] | 0.117 |
| Total bilirubin (mg/dl) | 0.50 [0.45, 0.80] | 0.50 [0.40, 0.70] | 0.50 [0.40, 0.80] | 0.60 [0.40, 0.80] | 0.183 |
| Amylase (U/L) | 16.00 [13.00, 19.50] | 19.00 [14.00, 23.50] | 15.00 [12.00, 19.00] | 19.00 [14.00, 24.00] | <0.001 |
| Alkaline phosphatase (U/L) | 131.00 [109.00, 139.00] | 117.00 [93.50, 138.50] | 123.50 [101.75, 152.50] | 117.00 [97.00, 144.00] | 0.257 |
| γ-Glutamyl-transferase (U/L) | 35.00 [15.50, 51.50] | 23.00 [14.00, 39.00] | 24.50 [16.00, 44.00] | 17.00 [12.00, 28.00] | <0.001 |
| Cholinesterase (U/L) | 6130.00 [5070.00, 6485.00] | 5770.00 [4750.00, 6600.00] | 6505.00 [5785.00, 7420.00] | 5570.00 [4690.00, 6490.00] | <0.001 |
| Creatine kinase (U/L) | 20.00 [16.50, 24.00] | 28.00 [20.00, 40.50] | 35.50 [22.75, 49.00] | 27.00 [19.00, 39.00] | 0.001 |
| Cortisol (mg/L) | 22.90 [16.30, 26.60] | 21.10 [17.25, 25.25] | 23.95 [16.68, 27.33] | 22.30 [18.10, 26.80] | 0.242 |
| Aldosterone (ng/L) | 85.00 [46.25, 180.75] | 85.00 [54.00, 141.00] | 85.00 [44.75, 139.50] | 71.50 [44.00, 114.00] | 0.006 |
| Renin (U/L) | 22.00 [14.00, 61.00] | 27.00 [14.00, 71.50] | 22.00 [10.00, 45.00] | 20.00 [10.00, 41.00] | 0.002 |
| Folic acid (µg/L) | 9.20 [5.90, 10.20] | 7.60 [5.80, 9.30] | 7.55 [5.88, 10.25] | 8.10 [6.20, 10.30] | 0.117 |
| Cotinine >15 µg/L = yes (%) | 3 ( 15.8) | 20 ( 10.9) | 30 ( 35.7) | 69 ( 13.6) | <0.001 |
| eGFR CKD-EPI* | 96.38 [81.20, 97.90] | 80.70 [66.42, 93.15] | 98.63 [88.61, 107.68] | 85.26 [71.72, 94.21] | <0.001 |
| Coronary artery disease (CAD) by angiographic status, n (%) |  |  |  |  |  |
| Normal (smooth contours) | 1 ( 5.3) | 36 ( 20.2) | 21 ( 25.6) | 80 ( 16.0) | 0.085 |
| Minor disease (11-49%) | 0 ( 0.0) | 12 ( 6.7) | 8 ( 9.8) | 50 ( 10.0) |  |
| 1 vessel disease (≥ 50%) | 5 ( 26.3) | 28 ( 15.7) | 17 ( 20.7) | 99 ( 19.8) |  |
| 2 vessel disease (≥ 50%) | 3 ( 15.8) | 48 ( 27.0) | 17 ( 20.7) | 104 ( 20.8) |  |
| 3 vessel disease (≥ 50%) | 10 ( 52.6) | 54 ( 30.3) | 19 ( 23.2) | 168 ( 33.5) |  |
| ≥ 10% max. stenosis | 18 ( 94.7) | 151 ( 84.8) | 65 ( 79.3) | 449 ( 89.6) | 0.026 |
| ≥ 20% max. stenosis | 18 ( 94.7) | 142 ( 79.8) | 61 ( 74.4) | 421 ( 84.0) | 0.061 |
| ≥ 50% max. stenosis | 18 ( 94.7) | 130 ( 73.0) | 53 ( 64.6) | 371 ( 74.1) | 0.051 |

* Kidney function was estimated using the Chronic Kidney Disease Epidemiology Collaboration (CKD-EPI) equation to calculate the glomerular filtration rate (eGFR).
